# Comparison of Algorithms for the Detection of Enteroviruses in Stool Specimens from Children Diagnosed with Acute Flaccid Paralysis

**DOI:** 10.1155/2017/9256056

**Published:** 2017-12-28

**Authors:** J. A. Adeniji, F. A. Ayeni, A. Ibrahim, K. A. Tijani, T. O. C. Faleye, M. O. Adewumi

**Affiliations:** ^1^Department of Virology, College of Medicine, University of Ibadan, Ibadan, Oyo State, Nigeria; ^2^WHO National Polio Laboratory, University of Ibadan, Ibadan, Oyo State, Nigeria; ^3^Faculty of Veterinary Medicine, University of Abuja, FCT, Abuja, Nigeria; ^4^Department of Microbiology, Faculty of Science, Ekiti State University, Ado-Ekiti, Ekiti State, Nigeria

## Abstract

This study was designed to compare both the cell culture dependent and independent enterovirus detection algorithms recommended by the WHO and assess how either might impact our perception of the diversity of enterovirus types present in a sample. Sixteen paired samples (16 isolates from RD cell culture and their corresponding stool suspension, i.e., 32 samples) from AFP cases in Nigeria were analyzed in this study. All the samples were subjected to RNA extraction, cDNA synthesis, the WHO recommended RT-snPCR, and its modification. Amplicons were sequenced and strains identified. Enterovirus diversity was the same between the isolates and fecal suspension for the control and five of the samples. It was, however, different for the remaining 10 (62.5%) samples. Nine (CV-B4, E6, E7, E13, E14, E19, E29, EV-B75, and EV-B77) and five (CV-A1, CV-A11, CV-A13, EV-C99, and PV2) EV-B and EV-C types, respectively, were detected. Particularly, E19 and EV-B75 were only recovered from the isolates while E14, EV-B77, CV-A11, and CV-A13 were only recovered from fecal suspension. Both the cell culture dependent and independent protocols bias our perception of the diversity of enterovirus types present in a sample. Hence, effort should be directed at harmonizing both for increased sensitivity.

## 1. Introduction

Enteroviruses (EVs) belong to genus* Enterovirus* in the family Picornaviridae and order Picornavirales. There are 13 species in the genus, and the type species of the genus is species C which has poliovirus as its best studied member [1]. EVs are nonenveloped viruses with icosahedral capsid symmetry and a diameter of 28–30 nM. The genome is an ~7.5 kb, single-stranded polyadenylated, positive-strand RNA with a covalently linked viral protein (VPg) at the 5′ terminus. The single open reading frame (ORF) in the genome is flanked by two untranslated regions (the 5′UTR and 3′UTR). The large polyprotein translated from the single ORF is processed to yield four structural proteins (VP1, VP2, VP3, and VP4) and seven nonstructural proteins. The sequence of the VP1 region has been correlated with EV serotype [2] and is now used for identification of EV types.

Most information on enterovirus diversity that has been made available in the last three decades has been courtesy of the Global Polio Eradication Initiative (GPEI). Hence, most of these EV isolates (polioviruses [PVs] and non-polio enteroviruses [NPEVs]) were recovered following the WHO recommended cell culture based enterovirus detection algorithm [3, 4]. With the goal (poliovirus eradication) of GPEI within reach, there is justifiable concern about facility associated escape of polioviruses into the community, after eradication [5]. Hence, as part of the endgame strategy, effort is ongoing to restrict poliovirus research in cell culture globally to few facilities (referred to as essential facilities) with the infrastructure to prevent and contain facility associated escape of the virus [5].

To facilitate implementation of this restriction in the near future, there has been significant motivation to develop very sensitive cell culture independent strategies for poliovirus (and other NPEVs) surveillance [6–8]. In line with this, a cell culture independent algorithm developed by Nix et al. [6] has been included in the recommended assays for enterovirus detection and identification by the WHO [9]. We recently showed [10] that this WHO recommended cell culture independent enterovirus detection algorithm [9] misses out enterovirus coinfection. This facilitates underestimation of a very common condition that was instrumental to the circulating vaccine derived poliovirus 2 (cVDPV2) outbreak [11] in Nigeria that lasted almost a decade. Consequently, we have described modification of the assay to expand its capacity, thereby facilitating detection and resolution of coinfection [10, 12].

In the light of the biases [13, 14] and limitations [10, 12] of both the cell culture dependent [4] and independent [9] algorithms, this study was designed to assess the impact of a switch from the former to the latter in the future. Further, it investigated how these algorithms alongside the coinfection (species) resolution assay impact our perception of the diversity of enterovirus types present in a sample. This study finds that both the cell culture dependent [4] and independent [9] algorithms have their strengths and weaknesses and unavoidably bias our perception of the diversity of enterovirus types present in a sample. It demonstrates the need to maximize the benefits of all available strategies in a bid to better describe the diversity of enteroviruses in any sample of interest. Finally, this study documents the first description of a Nigerian strain of EV-B77.

## 2. Methodology

### 2.1. Sample Collection

**Figure 1:**
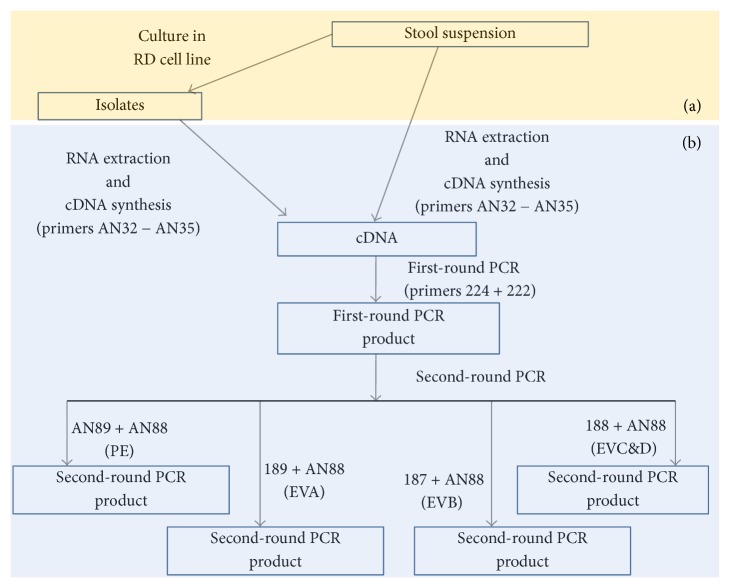
Schematic representation of the algorithm used in this study. (a) Sixteen RD cell culture isolates and their corresponding sixteen fecal suspensions were collected from the WHO National Polio Laboratory in Ibadan, Nigeria. (b) RNA was extracted from all thirty-two (32) samples (RD positive isolates and their corresponding suspension) and subsequently converted to cDNA. The cDNA was used as template in the 1st round PCR assay. The first-round PCR assay product was used as template in four different second-round PCR assays. Positive samples for the 2nd round PCR assays were sequenced and the result was used for enterovirus identification.

Sixteen RD positive isolates and their corresponding suspensions (making 32 samples in all, i.e., 16 pairs of isolates from cell culture and stool suspension) were analyzed in this study. The samples were collected from the WHO National Polio Laboratory in the Department of Virology, College of Medicine, University of Ibadan, Nigeria (subsequently referred to as the Polio Lab). Ten of the samples came from five cases (i.e., double stool samples collected at least 24 hours apart from the same case). The remaining six samples were single ones from six cases. One of these six samples was previously identified and confirmed by the Polio Lab as poliovirus 2 (PV-2). All the samples analyzed in this study were collected as part of the National Acute Flaccid Paralysis (AFP) surveillance programme. The samples were collected from children ≤ 15 years presenting with AFP between July and August 2015. The algorithm followed in this study is depicted in Figure 1.

### 2.2. RNA Extraction and cDNA Synthesis

RNA was extracted from isolates and suspensions independently using Jena Bioscience Total RNA extraction kit (Jena Bioscience, Jena, Germany) following the manufacturer's instructions. For cDNA synthesis, Jena Bioscience SCRIPT cDNA Synthesis Kit (Jena Bioscience, Jena, Germany) was used according to manufacturer's instructions. From the extract, 5.25 *μ*L of viral RNA was added to 4.75 *μ*L of cDNA synthesis mix. The 4.75 *μ*L of cDNA synthesis mix contained 2 *μ*L of SCRIPT RT buffer, 0.5 *μ*L of dNTP mix, 0.5 *μ*L DTT stock solution, 0.5 *μ*L of RNase inhibitor, 0.25 *μ*L of SCRIPT reverse transcriptase, and 0.25 *μ*L each primer AN32-AN35. The mixture was incubated at 42°C for 10 min followed by 50°C for 60 minutes in a Veriti thermal cycler (Applied Biosystems, California, USA).

### 2.3. Polymerase Chain Reaction

The 1st round PCR reaction (Figure 1) was a total of 30 *μ*L reaction. The reaction mix contained 6 *μ*L of Red Load Taq, 13.4 *μ*L of RNase free water, 0.3 *μ*L of primers 224 and 222, and 10 *μ*L of cDNA. Thermal cycling was done in a Veriti thermal cycler (Applied Biosystems, California, USA) as follows: 94°C for 3 minutes, then 45 cycles of 94°C for 30 seconds, 42°C for 30 seconds, and 60°C for 60 seconds, with ramp of 40% from 42°C to 60°C. This was then followed by 72°C for 7 minutes and held at 4°C until the reaction was terminated.

Four (PE-VP1-PCR, EA-VP1-PCR, EB-VP1-PCR, and EC-VP1-PCR [9]) different second-round PCR assays were run in this study (Figure 1). The 2nd-round PCR assay was also a 30 *μ*L reaction. The PCR reaction mix contained 6 *μ*L of Red Load Taq, 18.4 *μ*L of RNase free water, 0.3 *μ*L of forward and reverse primers, and 5 *μ*L of the first-round PCR product. Thermal cycling was done in a Veriti thermal cycler (Applied Biosystems, California, USA). The cycling conditions were 94°C for 3 minutes followed by 45 cycles of 94°C for 30 seconds, 42°C for 30 seconds, and extension at 60°C for 30 seconds, with ramp of 40% from 42°C to 60°C. This was then followed by 72°C for 7 minutes and subsequently held at 4°C until the reaction was terminated. The PCR products were resolved in a 2% agarose gels stained with ethidium bromide and viewed using a UV transilluminator.

### 2.4. Amplicon Sequencing

The amplicons of positive PCR reactions for the four second-round PCR assays were shipped to Macrogen Inc., Seoul, South Korea, where amplicon purification and sequencing were done. Sequencing was done using the respective forward and reverse primers for each of the four assays. Subsequently, using the enterovirus genotyping tool [15] and the sequence data, the enterovirus genotype and species were determined.

### 2.5. Nucleotide Sequences Accession Numbers

The sequences obtained from this study have been deposited in GenBank with accession numbers MF686545-MF686568.

### 2.6. Phylogenetic Analysis

The CLUSTAL **W** programme in MEGA 5 software [16] was used with default settings to align sequences of the enterovirus type(s) whose Nigerian strains were first described in this study alongside those retrieved from GenBank. Subsequently, a neighbor-joining tree was constructed using the same MEGA5 software [16] with the Kimura-2 parameter model [17] and 1000 bootstrap replicates. The accession numbers of sequences retrieved from GenBank for this analysis are indicated in the sequences name on the phylograms.

## 3. Results

### 3.1. Polymerase Chain Reaction (PCR) Assay

The expected ~330 bp fragment was successfully amplified for most of the assays carried out. For the* PE-VP1-PCR* screen, of the sixteen RD isolates subjected to this screen, 93.9% (15/16) were positive while 75.0% (12/16) of the corresponding suspensions were also positive. For the* EA-VP1-PCR* screen, 75.0% (12/16) of the RD isolates were positive as were 62.5% (10/16) of the corresponding suspensions. For the* EB-VP1-PCR* screen, 87.5% (14/16) and 68.8% (11/16) of the RD isolates and the corresponding suspensions were positive, respectively. Also, for the* EC-VP1-PCR* screen, 50% (8/16) and 37.5% (6/16) of the RD isolates and the corresponding suspension were positive, respectively (Table 1).

### 3.2. Enterovirus Genotyping

Of all the sixteen RD isolates, fifteen were amplified, successfully sequenced, and typed for the PE-VP1-PCR screen using the enterovirus genotyping tool. Their identities are as follows: E7 (3 isolates), E19 (2 isolates), E29 (1 isolate), EV B75 (1 isolate), CV A1 (1 isolate), E6 (2 isolates), E13 (4 isolates), and PV2 (1 isolate). For the EA-VP1-PCR screen, twelve RD isolates were successfully amplified but three were successfully typed and their identities are as follows: EV-C99 (1 isolate), CV-A1 (1 isolate), and PV2 (1 isolate). For the EB-VP1-PCR screen, fourteen RD isolates were successfully amplified, sequenced, and typed and their identities are as follows: E19 (2 isolates), E7 (3 isolates), E6 (2 isolates), E13 (4 isolates), E29 (1 isolate), EV-B75 (1 isolate), and CV-B4 (1 isolate). For the EC-VP1-PCR screen, nine RD isolates were amplified but two were successfully typed and their identities are PV2 (1 isolates) and EV-C99 (1 isolate) (Table 2). Over all, ten serotypes were identified for the RD isolates PCR screen comprising species B (70%) and species C (30%) (Table 3).

Of the corresponding 16 suspensions, twelve (12/16) were amplified but ten (10/16) were successfully sequenced and typed for the PE-VP1-PCR screen and their identities are as follows: E7 (1 strain), E13 (3 strains), E29 (1 strain), EV B77 (1 strain), CV A1 (1 strain), E6 (2 strains), and PV2 (1 strain). For the EA-VP1-PCR screen, eight suspensions were amplified, sequenced, and typed and their identities are as follows: EV-C99 (2 strains), CV-A11 (1 strain), CV-A13 (2 strains), CV-A1 (1 strain), PV2 (1 strain), and E29 (1 strain). For the EB-VP1-PCR screen, twelve were amplified but ten were successfully sequenced and typed, and their identities are as follows: E13 (3 strains), E14 (1 strain), E6 (2 strains), E7 (1 strain), CV-B4 (1 strain), E29 (1 strain), and EV-B77 (1 strain). For the EC-VP1-PCR screen, six suspensions were successfully amplified, sequenced, and typed and their identities are EV-C99 (2 strains), CV-A13 (2 strains), PV2 (1 strain), and CV-A1 (1 strain) (Table 2). Overall, twelve serotypes were identified for the suspension PCR screen comprising species B (58.3%) and species C (41.7%) (Table 3).

The enterovirus diversity was shown to be the same in the control (S/N 16) and 33.3% (5/15) of the samples analyzed (Table 2). To be precise, the diversity of enteroviruses was the same between RD cell culture isolates and fecal suspension for the control (S/N 16), Cases  4a, 5b, 6, 7, and 9 (Table 2). The diversity of enteroviruses was, however, different between RD cell culture isolates and fecal suspension for the remaining 66.7% (10/15) of the sample pairs analyzed (Table 2).

In summary, fourteen different enterovirus types were identified in this study. To be precise, nine (CV-B4, E6, E7, E13, E14, E19, E29, EV-B75, and EV-B77) and five (CV-A1, CV-A11, CV-A13, EV-C99, and PV2) EV-B and EV-C types, respectively, were detected in this study (Table 3). It is essential to emphasize that the single PV2 detected in this study was the control provided by the Polio Lab.

### 3.3. Phylogeny of EV-B77

**Figure 2:**
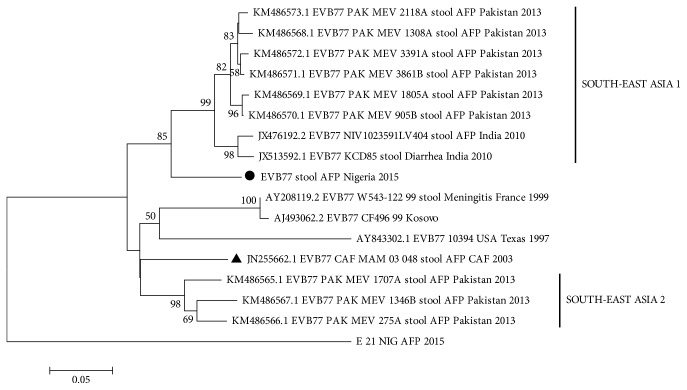
Phylogram of EV-B77. The phylogram is based on an alignment of partial VP1 sequences. The newly sequenced strains are highlighted with black circle. The strain previously recovered from Sub-Saharan Africa in 2003 is indicated with black triangle. The GenBank accession numbers of the strains are indicated in the phylogram. Bootstrap values are indicated if >50%.

This is the first EV-B77 strain described in Nigeria and the second in Sub-Saharan Africa till date. The topology of the phylogenetic tree suggests that the EV-B77 detected in this study is different from all that has been described till date. More importantly, it is different from the only Sub-Saharan Africa strain described till date which was recovered in Central Africa Republic in 2003 (Figure 2).

## 4. Discussion

### 4.1. Direct Detection from Clinical Specimen versus after Culture in RD Cell Line

From this study, it was observed that more enteroviruses were detected per sample by the PE-VP1-PCR assay after the suspension had been subjected to culture in RD cell line. For example, Case  1b (Table 2) isolate was identified as E19 while there was no evidence of enterovirus presence in the corresponding suspension. In the same light, the isolates of Cases  4b and 5a (Table 2) were identified as E13 while there was also no evidence of enterovirus presence in their corresponding suspensions. Considering enteroviruses were detected in both the isolate and stool suspensions of other samples and even the ≥24-hour pair of some of the samples in questions, it is unlikely that the observation is due to the presence of nonspecific inhibitors of PCR. Rather, this finding suggests that, in the fecal suspension, the virus titre might have been too low (i.e., below the detection limit of the assay) to be detected directly. However, RD cell culture appeared to increase the virus titre to a level that was subsequently detectable by the PE-VP1-PCR assay. This thereby validates the value and use of cell culture for enterovirus detection and identification as it significantly increases virus titre and thereby enhances our capacity to detect and identify the virus types present.

It is however important to note that though some EV types were recovered in both the fecal suspension and RD cell culture, some types appear to be specifically recovered in each detection algorithm (Table 3). The enterovirus diversity was shown to be the same between RD cell culture isolates and fecal suspension for the control and 5/15 (Cases  4a, 5b, 6, 7, and 9) of the sample pairs analyzed. It was, however, different for the remaining 10/15 (66.7%) sample pairs analyzed. Particularly fascinating is the observation that in some instances the enterovirus isolate detected by the PE-VP1-PCR assay in RD cell culture supernatant is different from what was detected in the corresponding suspension. For example, in Case  9 (Table 2), the isolate was identified as EVB75 while EVB77 (first detection in Nigeria) was identified in the corresponding suspension. Also, in Case  1a (Table 2) where the isolate was identified as E19, E13 was detected in the corresponding stool suspension, despite the fact that it is well known [18, 19] and also documented in this study (Table 2) that RD cell line is both susceptible and permissive to E13. Hence, if the most abundant genome was selectively detected in the above stated instances, these discrepancies suggest that in either case the most abundant genome in the suspension was different from that in the cell culture supernatant. This therefore confirms that culture in RD cell line selectively amplifies one enterovirus genome over the other in cases of coinfection [13, 14, 20], even in cases where both enterovirus types belong to the same species (Table 2). Should this observation be a valid biological phenomenon, its biological basis might further illuminate how culture of enteroviruses in RD cell line influences our perception of the serotype diversity in a sample. Furthermore, this might also indicate that the dynamics of enterovirus culture in RD cell line might not be representative of what happens in the intestinal tract and consequently should not be represented as such.

### 4.2. The Impact of Mixture Resolving Assays

Cases of enterovirus coinfection were established in 53.3% (EV-B/C = 40%: EV-B = 20%) of the samples analyzed in this study. It is however worthy of note that in these coinfected samples (Table 2), the enterovirus types identified with the PE-VP1-PCR assay were mainly EV-Bs while the EV-C coinfection was majorly detected by the species-specific assays. The only exception was in Case  9, where, in both the fecal suspension and the RD cell culture isolate, the enterovirus type detected using the PE-VP1-PCR assay was CVA1 while the EVB-VP1-PCR assay detected CVB4 (Table 2). The perceived predilection of the PE-VP1-PCR assay for EV-Bs is not because the primers used for the assay have a bias for EV-Bs. In fact, similar studies using the same assay directly on fecal suspensions without culture in RD cell line show an abundance of EV-As [21], while those where the same assay was used directly on fecal suspensions that did not show CPE in RD cell line showed an abundance of EV-Cs [12]. Hence, the predominance of EV-Bs as documented by the PE-VP1-PCR assay, in this study, might be due to the fact that only samples that had yielded isolate in RD cell lines were selected and analyzed in this study. Considering the EV-B bias [13, 14, 20] of RD cell line, this might not be surprising. This observation however suggests that in cases of coinfection involving different enterovirus species, the chances of detecting all the enterovirus present in the sample will be more likely enhanced by the addition of species-specific primers to the PCR protocols. For members of the same species, however, combining cell culture with direct detection from the specimen might be the strategy of choice (Table 2).

### 4.3. The Value of Paired Samples

The need and value of collecting two stool samples (paired samples) about 24 hours apart from any AFP case are well entrenched in the GPEI enterovirus detection protocols [4, 9]. The results of this study further emphasize the importance of this principle for enterovirus surveillance. For example, it was observed that E13 and E19 were detected in Case  1a but only E19 was identified in Case  1b. Also, while E7 and EVC99 were detected in Case  2a, E14 in addition to E7 and EVC99 were detected in Case  2b. More importantly, E13 was detected in Case  5a, while no enterovirus was detected in Case  5b (Table 2). The results of this study therefore further demonstrate that, without paired samples, many enterovirus infections would be missed. Consequently, we recommended that this principle be implemented for enterovirus surveillance in general and not just for AFP surveillance.

### 4.4. Enterovirus Detection Algorithms and the Risk of Facility Associated Escape of Poliovirus after Containment

We have shown that both the cell culture dependent [4] and independent [9] protocols recommended by the WHO for enterovirus detection unavoidably bias our perception of the diversity of enterovirus types present in a sample (Tables 2 and 3). We have also shown the shortcomings of a Pan-Enterovirus RT-PCR detection assay which is predicated on the false assumption that coinfections are not significant when enterovirus infections are being considered. Though the anticipated need to prevent the risk of facility associated escape of polioviruses cannot be overemphasized, the findings of this study suggest that enterovirologists should attempt to maximize the benefits of available strategies in a bid to better describe the diversity of enteroviruses in any sample of interest.

On the other hand, effort should be put into expanding the species [22] or serotype [23] specific nextgen sequencing strategies that have already been developed to accommodate other enterovirus types and species. They also have to be expanded to go beyond using isolates recovered from cell culture to direct detection from clinical specimen. Such development might facilitate a successful switch from cell culture dependent to independent strategies without necessarily losing out on breadth and sensitivity.

## Figures and Tables

**Table 1:** Results of the RT-semi-nested PCR assays done in this study.

Sample		Isolates					Suspension				
		Species specific assays					Species specific assays				
S/N	Cases	PE	EV A	EV B	EV C&D	Summary	PE	EV A	EV B	EV C&D	Summary
1	Case 1a	+	+	+	+	++++	+	+	+	−	+++
2	Case 1b	+	−	+	−	++	−	−	−	−	

3	Case 2a	+	+	+	−	+++	+	+	+	+	++++
4	Case 2b	+	+	+	+	++++	+	+	+	+	++++

5	Case 3a	+	+	+	+	++++	+	+	+	−	+++
6	Case 3b	+	+	+	+	++++	+	+	+	+	++++

7	Case 4a	+	−	+	+	+++	+	−	+	−	++
8	Case 4b	+	+	+	−	+++	−	−	−	−	

9	Case 5a	+	+	+	−	+++	−	−	−	−	
10	Case 5b	−	−	−	−		−	−	−	−	

11	Case 6	+	+	+	−	+++	+	+	+	−	+++

12	Case 7	+	+	+	+	++++	+	+	+	−	+++

13	Case 8	+	−	+	−	++	+	−	+	−	++

14	Case 9	+	+	+	+	++++	+	+	+	+	++++

15	Case 10	+	+	+	+	++++	+	+	+	+	++++

16	Control	+	+	−	+	+++	+	+	−	+	+++

*Total summary*		*15*	*12*	*14*	*9*		*12*	*10*	*11*	*6*	

**Table 2:** Results of nucleotide sequencing and identification of enterovirus isolates and strains recovered in this study.

Sample		Isolates					Suspension					Summary of serotypes	Species
		Species specific assays					Species specific assays						
S/N	Cases	PE	EV A	EV B	EV C&D	Serotype identification	PE	EV A	EV B	EV C&D	Serotype identification		
1^*∗∗*^	Case 1a	E19	NU	E19	NU	E19	E13	NU	E13		E13	E13, E19	EV-B^*∗*^
2	Case 1b	E19		E19		E19						E19	EV-B

3	Case 2a	E7	EV C99	E7		E7, EV-C99	NU	EV C99	NU	EV C99	EV-C99	E7, EV-C99	EV-B, EV-C^*∗*^
4	Case 2b	E7	NU	E7	EV C99	E7, EV-C99	NU	EV C99	E14	EV C99	E14, EV-C99	E7, E14, EV-C99	EV-B, EV-C^*∗*^

5	Case 3a	E6	NU	E6	NU	E6	E6	CV A11	E6		E6, CV-A11	E6, CV-A11	EV-B, EV-C^*∗*^
6	Case 3b	E6	NU	E6	NU	E6	E6	CV A13	E6	CV A13	E6, CV-A13	E6, CV-A13	EV-B, EV-C^*∗*^

7	Case 4a	E13		E13	NU	E13	E13		E13		E13	E13	EV-B
8	Case 4b	E13	NU	E13		E13						E13	EV-B

9^*∗∗*^	Case 5a	E13	NU	E13		E13						E13	EV-B
10	Case 5b												

11	Case 6	E7	NU	E7		E7	E7	NU	E7		E7	E7	EV-B

12	Case 7	E29	NU	E29	NU	E29	E29	E29	E29		E29	E29	EV-B

13	Case 8	EV B75		EV B75		EV-B75	EV B77		EV B77		EV-B77	EV-B75, EV-B77	EV-B^*∗*^

14	Case 9	CV A1	CV A1	CV B4		CV-A1, CV-B4	CV A1	CV A1	CV B4	CV A1	CV-A1, CV-B4	CV-A1, CV-B4	EV-B, EV-C^*∗*^

15	Case 10	E13	NU	E13	NU	E13	E13	CV A13	E13	CV A13	E13, CV-A13	E13, CV-A13	EV-B, EV-C^*∗*^

16^*∗∗∗*^	Control	PV 2	PV 2		PV 2	PV2	PV 2	PV 2		PV 2	PV2	PV2	EV-C

NU: not usable; **∗**: coinfection.

**Table 3:** Enterovirus types identified in this study.

*Enterovirus* species	Isolate		Suspension		Total *Enterovirus* types
	*Enterovirus* types	Number of types (%)	*Enterovirus* types	Number of types (%)	
EV-B	E6, E7, E13, *E19*, E29, *EV-B75*, CV-B4	7 (70%)	E6, E7, E13, *E14,* E29, *EV-B77*, CV-B4	7 (58.3%)	9 (64.3%)
EV-C	EV-C99, CV-A1, PV-2^*∗*^	3 (30%)	EV-C99, CV-A1, *CV-A11, CV-A13,* PV-2^*∗*^	5 (41.7%)	5 (35.7%)

Total		10 (100%)		12 (100%)	14 (100%)

E: echovirus, EV: enterovirus, CV: coxsackievirus, PV: poliovirus, and *∗*: control PV2; italics: viruses that were peculiar to the different detection algorithms.
